# Sensitive quantification of BB-22 and its metabolite BB-22 3-carboxyindole, and characterization of new metabolites in authentic urine and/or serum specimens obtained from three individuals by LC–QTRAP-MS/MS and high-resolution LC–Orbitrap-MS/MS

**DOI:** 10.1007/s11419-018-0448-3

**Published:** 2018-10-16

**Authors:** Kayoko Minakata, Koutaro Hasegawa, Hideki Nozawa, Itaru Yamagishi, Takeji Saitoh, Atsuto Yoshino, Masako Suzuki, Takuya Kitamoto, Osamu Suzuki, Kanako Watanabe

**Affiliations:** 1 Department of Legal Medicine, Hamamatsu University School of Medicine, 1-20-1 Handayama, Higashi-ku, Hamamatsu, 431-3192 Japan; 2 Department of Emergency and Disaster Medicine, Hamamatsu University School of Medicine, 1-20-1 Handayama, Higashi-ku, Hamamatsu, 431-3192 Japan; 3 Advanced Research Facilities and Services, Hamamatsu University School of Medicine, 1-20-1 Handayama, Higashi-ku, Hamamatsu, 431-3192 Japan

**Keywords:** BB-22, BB-22 3-carboxyindole, In vivo metabolites in human urine, QTRAP mass spectrometry, High-resolution mass spectrometry, Authentic serum and urine specimens

## Abstract

**Purpose:**

A synthetic cannabinoid BB-22 and its metabolite BB-22 3-carboxyindole have not yet been quantified in human urine. The aim of this study is to establish a sensitive analytical method for the quantification of BB-22 and its 3-carboxyindole in human serum and urine specimens, and the characterization of the unreported metabolites of BB-22 in authentic urine specimens from three individuals.

**Methods:**

These compounds were extracted from β-glucuronide-hydrolyzed and unhydrolyzed urine and/or serum via liquid–liquid extraction. The identification and quantification were performed using liquid chromatography (LC)–QTRAP-tandem mass spectrometry (MS/MS) and the characterization of the new metabolites was made by high-resolution LC–MS/MS.

**Results:**

The limits of detection of BB-22 and BB-22 3-carboxyindole were 3 and 30 pg/mL in urine, respectively. The devised method was applied to quantify these compounds in authentic serum and urine obtained from two drug abusers and in urine from one drug abuser. The serum levels of BB-22 were 149 and 6680 pg/mL, and those of BB-22 3-carboxyindole were 0.755 and 38.0 ng/mL in cases 1 and 2, respectively. The urine levels of BB-22 were 5.64, 5.52 and 6.92 pg/mL and those of BB-22 3-carboxyindole were 0.131, 21.4 and 5.15 ng/mL in cases 1, 2 and 3, respectively. New monohydroxyl metabolites retaining the structure of BB-22 were found in the urine specimens.

**Conclusions:**

The synthetic cannabinoid BB-22 and its metabolite BB-22 3-carboxyindole were identified and quantified in authentic human serum and urine specimens for the first time, and new metabolites of BB-22 were tentatively identified in authentic urine specimens obtained from three drug users in this study.

## Introduction

**Fig. 1:**
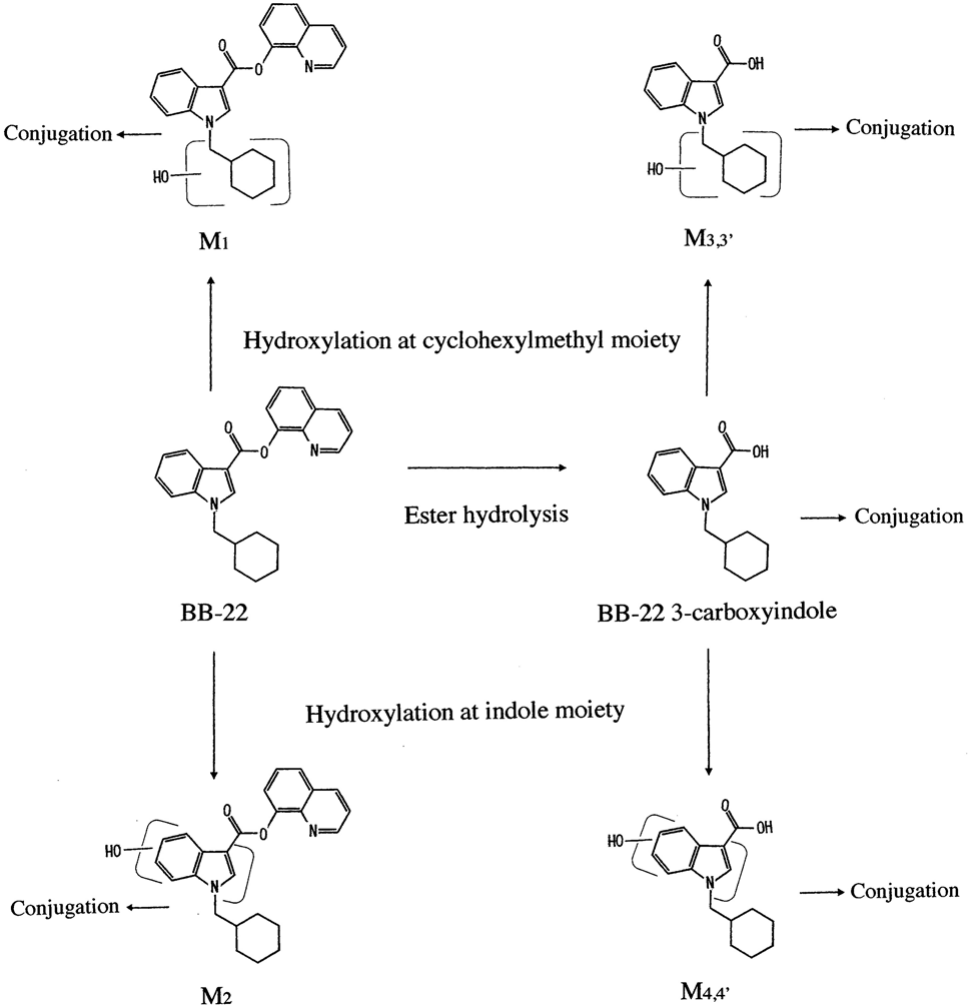
Structures of BB-22, its metabolites BB-22 3-carboxyindole and metabolites M1–M4,4′ showing their metabolic pathways

A psychotropic synthetic cannabinoid (SC) BB-22 [quinolin-8-yl 1-(cyclohexylmethyl)-1*H*-indole-3-carboxylate], shown in Fig. 1, was firstly identified in herbal-type illegal products in 2013 [1]. The first generation SC, JWH-018, was active toward the CB_1_ receptor having an affinity 4.5 times higher than that of ∆^9^-tetrahydrocannabinol contained naturally in *Cannabis sativa*, while the affinity of BB-22 was reported to be about 30 times higher than that of JWH-018 [2]. As to the concentrations of BB-22 in any authentic human specimens, one study reported on its plasma level, 97 pg/mL, based on liquid chromatography (LC)–mass spectrometry (MS) in 2015 [3], but its urine level has not yet been reported. BB-22 3-carboxyindole [1-cyclohexylmethyl-1*H*-indole-3-carboxylate] and its hydroxylated metabolites (M3,3′ and M4,4′), shown in Fig. 1, were reported to be produced after in vitro hydrolysis by carboxylesterases [4] and after hepatocyte in vitro metabolization [5]. However, these metabolites were also produced from MDMB-CHMICA and ADB-CHMICA [5]. The other metabolites retaining the structure of BB-22 have not been identified yet, even via in vitro study.

In the present study, BB-22 and BB-22 3-carboxyindole in authentic human urine specimens have been quantified for the first time by a sensitive LC–QTRAP tandem mass spectrometry (MS/MS) technique that was used almost in the same way for 5F-PB-22 and other six SCs in human urine specimens [6, 7] and 5F-NNEI in human serum and urine specimens [8]. The present serum and urine specimens were obtained from two living patients presenting to emergency department of our university, and a urine specimen from one individual who was suspected by the police of having used illegal drugs. Although these specimens were collected in 2013, we could not identify the SC at that time mainly due to minute amounts of the SC in the unchanged forms and lower sensitivity of the conventional LC–MS/MS instrument used at that time. The new metabolites of BB-22 after monohydroxylation (M1 and M2 shown in Fig. 1) and metabolites after ester hydrolysis with monohydroxylation (M3,3′ and M4,4′) were tentatively identified in urine specimens using LC–QTRAP-MS/MS and high-resolution LC–Orbitrap-MS/MS.

## Materials and methods

### Materials

BB-22, BB-22 3-carboxyindole and AB-PINACA were obtained from Cayman Chemical (Ann Arbor, MI, USA); β-glucuronidase-type-H-1 from Sigma (St. Louis, MO, USA); methanol and acetonitrile suitable for LC–MS, 1-chlorobutane (CB) suitable for amino acid analysis and other chemicals of analytical grade from Wako Pure Chemical Industries (Osaka, Japan). Pure water with a specific resistance of 18 MΩ cm was used (Millipore, Bedford, MA, USA).

Serum and urine specimens from healthy subjects, under their permission with informed consent, were used as blank samples, and those spiked with several amounts of BB-22 and BB-22 3-carboxyindole were used as quality control samples. The authentic serum and urine specimens in all three cases were stored at −80 °C until analyses.

### Cases

In cases 1 and 2, serum and urine specimens were collected from two patients at the emergency department of our university hospital in July 2013. In case 3, the urine specimen was collected by police in August 2013.

### Standard solutions

Individual stock solutions of BB-22 and BB-22 3-carboxyindole were prepared separately by dissolving appropriate amounts of each compound in acetonitrile at 0.1 mg/mL and stored at −30 °C. Working calibration solutions and quality control solutions were prepared daily by diluting the stock solutions with blank serum or urine at 5 pg–20 ng/mL. AB-PINACA at 1 ng/mL in serum or urine was used as internal standard (IS) for the quantification of BB-22 and BB-22 3-carboxyindole.

### Pretreatment

The hydrolysis and extraction procedures were described in our previous studies for urine [6–8]. In the case of serum, a 100-μL aliquot of sample was added with two stainless beads (3-mm diameter) and 300 μL of 0.1 M acetate buffer (pH 5). Here, 25 μL of β-glucuronidase solution (type H-1, 25,000 unit) was added and incubated at 37 °C for 2 h in the case of a β-glucuronide-hydrolyzed sample; this step was skipped in the case of the unhydrolyzed sample. To the sample, 1 μL of 100 pg AB-PINACA as IS and 750 μL of CB were added, and vortexed for 30 s. Then 40 mg of CH_3_COONa and 160 mg of MgSO_4_ were added, further vortexed for 90 s, and then centrifuged at 10,000 *g* for 4 min. The upper CB layer was transferred to a new tube. To the aqueous layer, 650 μL of CB was added again, vortexed for 60 s and centrifuged at 10,000 *g* for 4 min, and the CB layer was collected. The combined CB layer was evaporated to near-dryness at room temperature using a centrifugal dryer (miVac Duo LV; Genevac Ltd, Ipswich, England). The residue was reconstituted in 100 μL of methanol, and centrifuged at 10,000 *g* for 60 s. The supernatant was used for the analysis by LC–MS/MS.

### Instrumental conditions

LC–MS/MS was performed on a 4000 QTRAP MS/MS system (AB SCIEX, Framingham, MA, USA) in the positive ion mode. LC was performed using an Acquity instrument (Waters, Milford, MA, USA). A filter named SUMIPAX Filter PG-ODS (Sumika Chemical Analysis Service, Osaka, Japan) was attached before LC separation. The LC column for the chromatographic separation was TSK-GEL ODS-100 V (150 × 2.0 mm i.d., particle size 5 μm; Tosoh, Tokyo, Japan). The mobile phase consisting of 35% B (i.e., 65% A) was set at a flow-rate of 200 μL/min for 2 min and then gradient elution was performed using 35–65% B over 10 min, switched to 100% B, held for 2 min, and returned to initial conditions over 8 min, where solvent A was pure water containing 0.1% formic acid and 10 mM ammonium acetate, and solvent B was 100% methanol. The MS/MS conditions were: ion source temperature, 700 °C; spray needle voltage, + 5.5 kV; sheath gas pressures, 30 units for gas 1 and 50 units for gas 2; curtain gas flow, 50 units. The tandem MS collision energies and ion transitions were: 21 eV and *m/z* 385 → 214 for BB-22, 29 eV and *m/z* 258 → 118 for BB-22 3-carboxyindole, and 35 eV and *m/z* 331 → 215 for AB-PINACA (IS), respectively. A 5-μL aliquot of the final extract solution was injected into the LC–MS/MS instrument.

LC–high-resolution-MS/MS was performed on an Ulti Mate 3000 coupled to a Thermo Scientific QExactive (quadrupole-Orbitrap) mass spectrometer (Thermo Scientific, Waltham, MA, USA). Chromatographic separation was achieved with the same column and the same solvent conditions as described above for LC–MS/MS performed on a 4000 QTRAP MS/MS system. The QExactive mass spectrometer was operated in positive ionization mode. The MS or MS/MS conditions were: spray voltage, 3.5 kV; capillary temperature, 250 °C; heater temperature, 350 °C; sheath gas, flow rate 50 units and auxiliary gas, flow rate 15 units. Nitrogen was used for the collision-induced dissociation experiment. The instrument was calibrated every 24 h. The full MS resolution was 70,000 with scan range of *m/z* 220–2000 and MS/MS resolution was 17,500 with scan range of *m/z* 50–2000. A 5-μL aliquot of the final extract solution was injected into the instrument.

## Results and discussion

### Selected reaction monitoring chromatograms and product ion spectra

**Fig. 2:**
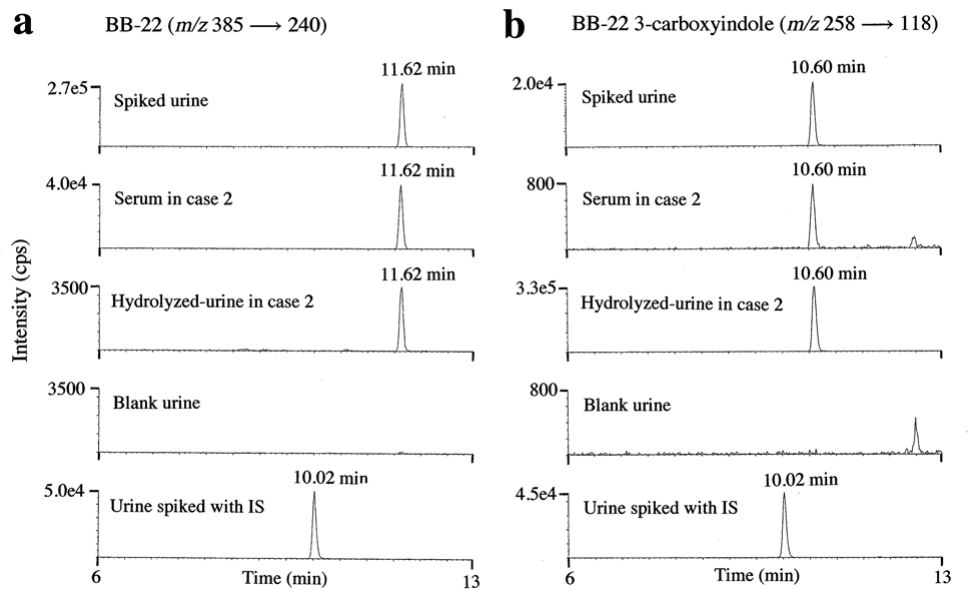
Selected reaction monitoring (SRM) chromatograms by liquid chromatography (LC)–QTRAP tandem mass spectrometry (MS/MS) for the detection of BB-22 (**a**), where the extracts from blank urine spiked with the reference standard at 1.0 ng/mL, the serum in case 2, the β-glucuronide-hydrolyzed urine in case 2, the blank urine and the blank urine spiked with the internal standard (IS) at 1.0 ng/mL are shown from the top to the bottom panel. The equivalent SRM chromatograms for the detection of BB-22 3-carboxyindole (**b**) for the blank urine spiked with the reference standard at 10 ng/mL, the serum in case 2, the hydrolyzed urine in case 2, the blank urine and the blank urine spiked with the IS at 1.0 ng/mL are shown. The collision energies for BB-22 and its 3-arboxyindole were 21 and 29 eV, respectively

The selected reaction monitoring (SRM) chromatograms by LC–MS/MS are shown for the detection of BB-22 (Fig. 2a), where the extract from blank urine spiked with the reference standard at 1.0 ng/mL, the extract from serum in case 2, the extract from β-glucuronide-hydrolyzed urine in case 2, the extract from blank urine and the extract from blank urine spiked with IS at 1.0 ng/mL are shown from the top to the bottom. The equivalent SRM chromatograms are also shown for BB-22 3-carboxyindole (Fig. 2b), for the blank urine spiked with the reference standard at 10 ng/mL, the serum in case 2, β-glucuronide-hydrolyzed urine in case 2, blank urine and the blank urine spiked with IS at 1.0 ng/mL. Each protonated molecular ion was used as the precursor ion for acquisition of SRM chromatograms.

**Fig. 3:**
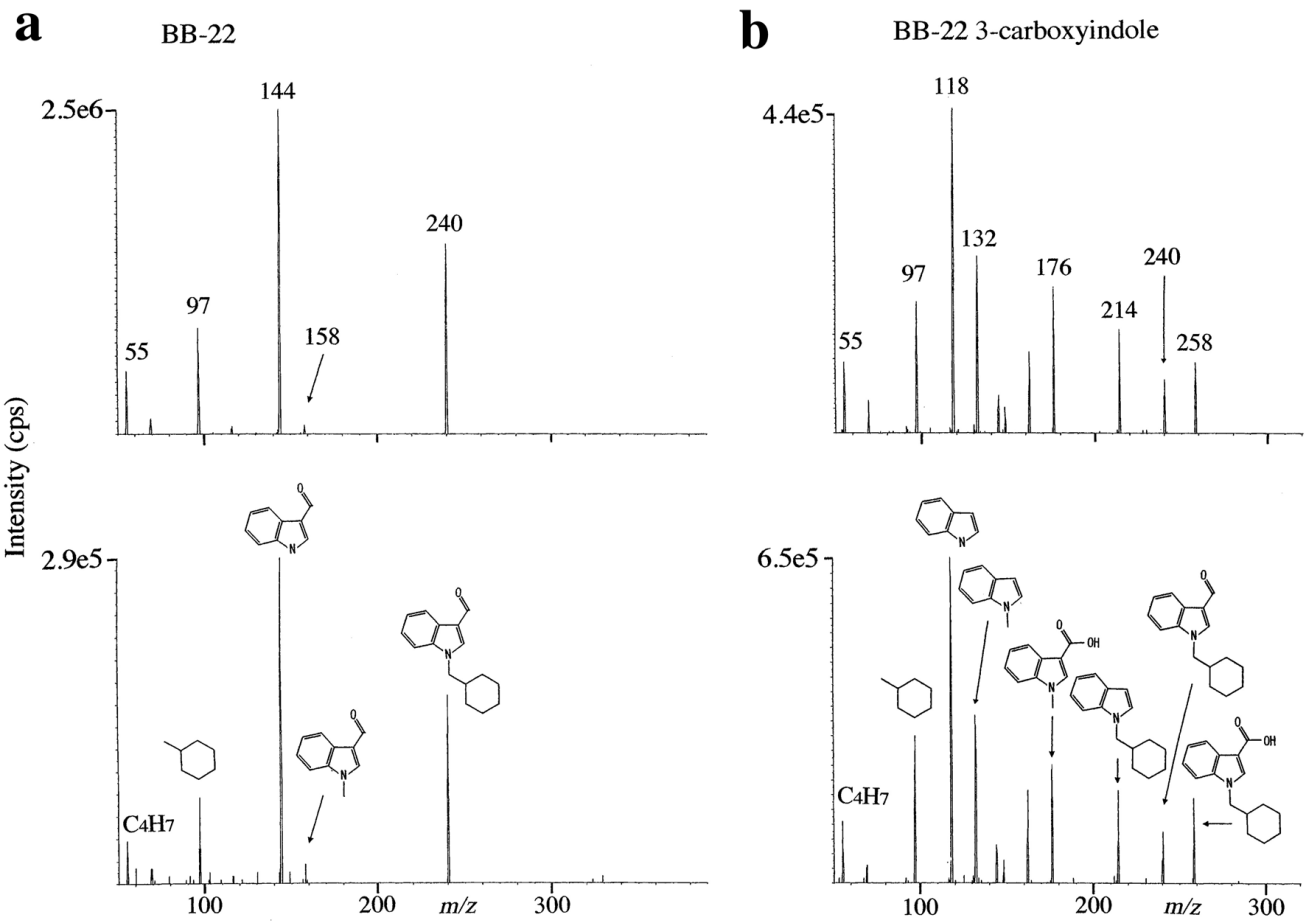
Product ion spectra for the detection of BB-22 (**a)**, where the reference standard at 10 ng/mL in methanol is shown in the upper panel and the extract from serum in case 2 in the lower panel. Product ion spectra for BB-22 3-carboxyindole (**b)**, where the reference standard at 100 ng/mL in methanol is shown in the upper panel and the extract from hydrolyzed urine in case 2 in the lower panel. The collision energies for BB-22 and its 3-arboxyindole metabolite were 51 and 29 eV, respectively

Figure 3a shows the product ion spectrum obtained from the reference standard of BB-22 at 10 ng/mL in methanol in the upper panel and that from the serum in case 2 in the lower panel for unequivocal identification, where the collision energy at 51 eV was adopted in the detection because the collision energy at 21 eV, though suitable for quantification, did not give the enough numbers of qualifier ions. Figure 3b shows the equivalent product ion spectrum obtained from the reference standard of BB-22 3-carboxyindole at 100 ng/mL in methanol and that from hydrolyzed urine in case 2, except for the collision energy at 29 eV used.

The relative peak height ratios of principal product ions (signal-to-noise ratio > 3) derived from the respective reference standard protonated molecular ions and those from serum and urine samples are listed in Table 1 by taking the highest product ions to be 100. The ratios of the reference standard of BB-22 and BB-22 3-carboxyindole and those of serum and urine samples almost agreed with one another, confirming that the peaks from the samples in Fig. 2 were due to the target compounds.

**Table 1:** Relative intensities of the product ions of BB-22 and BB-22 3-carboxyindole derived from the reference standard solutions and from serum and urine in case 2

Compound (collision energy) and sample	Protonated molecular ion (*m/z*)	Product ion (*m/z*) Percent product ion intensity				
BB-22 (51 eV)	(385.2)	(144.1)	(240.1)	(97.1)	(55.0)	
RS (10 ng/mL)		100	58.1	31.7	19.0	
Serum in case 2		100	57.8	26.8	13.4	
Tenfold concentrated urine in case 2		100	53.6	33.9	19.6	
BB-22 3-carboxyindole (29 eV)	(258.1)	(118.1)	(132.1)	(97.1)	(176.1)	(214.1)
RS (100 ng/mL)		100	51.8	45.1	36.6	28.1
Serum in case 2		100	53.1	43.8	42.1	27.7
Hydrolyzed urine in case 2		100	53.5	40.1	45.8	31.7

*RS* reference standard

### Validation of the method

The validation experiments were performed using the unhydrolyzed samples, because these compounds were stable during β-glucuronidase hydrolysis, as mentioned in the previous report on 5F-PB-22 having ester linkage [5].

The linearity of BB-22 using the present method was examined by spiking the compound (at 0, 10, 20, 60, 200 or 2000 pg/mL in serum and at 0, 5, 10, 30, 100 or 1000 pg/mL in urine) to blank matrices (*n* = 6 at each concentration). The linearity of BB-22 3-carboxyindole was examined by spiking the compound (at 0, 0.2, 0.6, 2 or 20 ng/mL in serum and at 0, 0.1, 0.3, 1 or 10 ng/mL in urine, *n* = 6 each). The regression equations for the calibration curves are listed in Table 2, where the correlation coefficients were 0.990–0.999. The limits of detection (signal-to-noise ratio = 3) of BB-22 and BB-22 3-carboxyindole were 3 and 30 pg/mL in urine, and 6 and 60 pg/mL in serum, respectively.

**Table 2:** Regression equations, correlation coefficients and limits of detection of BB-22 and BB-22 3-carboxyindole spiked into blank serum and urine detected by liquid chromatography–tandem mass spectrometry

Compound	Range	Equation	Correlation coefficient	Limit of detection (pg/mL)
BB-22 in serum	10–2000 pg/mL	*y *= 0.00543 *x* + 0.0096	0.997	6
BB-22 in urine	5–1000 pg/mL	*y *= 0.00647 *x* + 0.0112	0.990	3
BB-22 3-carboxyindole in serum	0.2–20 ng/mL	*y *= 0.000226 *x* + 0.00107	0.999	60
BB-22 3-carboxyindole in urine	0.1–10 ng/mL	*y* = 0.000240 *x* + 0.000965	0.997	30

The precisions and the accuracies were assessed by analyzing samples spiked with BB-22 at 10, 20, 60, 200 and 2000 pg/mL in serum and at 5, 10, 30, 100 and 1000 pg/mL in urine, respectively, three times a day as well as on three different days. In the determination of precisions and the accuracies of BB-22 3-carboxyindole, samples were spiked with it at 0.2, 0.6, 2 and 20 ng/mL in serum and at 0.1, 0.3, 1 and 10 ng/mL in urine, respectively. The accuracy data were 82.6–124% and the precision data were not greater than 28.3% for intraday and interday measurements as listed in Table 3. These data could be considered to be generally within the acceptable range for the quantification.

**Table 3:** Intraday and interday accuracy/precision, recovery and matrix effect data of BB-22 and BB-22 3-carboxyindole spiked into blank serum and urine (*n* = 3 each)

Concentration spiked	Intraday		Interday^a^		Recovery (%)	Matrix effect (%)
	Accuracy (%)	Precision (%)	Accuracy (%)	Precision (%)		
BB-22 in serum (pg/mL)						
10	104	21.1	114	14.7	75.1	87.6
20	89.5	9.5	95.7	8.3	71.1	75.0
60	96.3	2.6	102	3.9	56.7	81.2
200	105	3.6	109	11.1	76.1	94.4
2000	102	2.8	109	13.2	72.2	94.7
BB-22 in urine (pg/mL)						
5	99.0	20.2	93.0	8.4	77.9	108
10	117	7.7	106	9.1	105	82.4
30	94.4	4.3	91.0	1.6	101	80.4
100	111	12.2	115	15.6	99.8	80.0
1000	110	8.4	107	16.5	82.4	94.7
BB-22 3-carboxyindole in serum (ng/mL)						
0.2	100	14.6	100	7.5	81.0	82.6
0.6	101	13.9	94.4	13.6	56.9	102
2	104	0.9	100	5.7	105	106
20	97.1	3.2	101	3.4	69.2	98.2
PB-22 3-carboxyindole in urine (ng/mL)						
0.1	114	28.3	124	18.7	103	87.1
0.3	103	26.3	102	2.7	81.7	107
1	82.6	7.3	95.6	9.7	75.0	95.0
10	100	6.8	104	0.8	80.9	101

^a^The interday experiment was made with triplicate determinations × 3 days

The extraction recovery and the matrix effect were calculated according to the method described [5, 6]. The recoveries in the quantification ranges were 56.7–105% (*n* = 3 at each concentration) and the matrix effects were 75.0–108% (*n* = 3 at each concentration) as listed in Table 3, which is acceptable for quantitative analysis.

### Quantification of unchanged BB-22 and BB-22 3-carboxyindole in authentic serum and urine specimens

Samples were diluted with blank matrices when the levels of target compounds were out of quantification range. The levels of BB-22 and BB-22 3-carboxyindole in unhydrolyzed serum, those in β-glucuronide-hydrolyzed serum, those in unhydrolyzed urine and those in hydrolyzed urine were measured (*n* = 3 each), and the results are summarized in Table 4, where the increment of the compound by hydrolysis could be estimated roughly as the concentration of its glucuronidated form. The levels of BB-22 3-carboxyindole in urine increased to 1.7–118 times after the hydrolysis.

**Table 4:** Quantifications of BB-22 (pg/mL) and BB-22 3-carboxyindole (ng/mL) in unhydrolyzed and hydrolyzed serum and/ or urine, and their increments by hydrolysis (i.e., glucuronide compounds) for three cases, respectively

Case	Concentration		
	1	2	3
BB-22 in serum (pg/mL)			
Unhydrolyzed serum	149 ± 16	6680 ± 420	–^a^
Hydrolyzed serum	192 ± 14	6110 ± 780	–^a^
BB-22 in urine (pg/mL)			
Unhydrolyzed urine	5.64 ± 0.36	5.52 ± 0.50	6.92 ± 0.42
Hydrolyzed urine	6.22 ± 1.18	5.86 ± 0.98	7.14 ± 0.76
BB-22 3-carboxyindole in serum (ng/mL)			
Unhydrolyzed serum	0.755 ± 0.134	38.0 ± 1.1	–^a^
Hydrolyzed serum	0.679 ± 0.110	46.2 ± 1.8	–^a^
BB-22 3-carboxyindole in urine (ng/mL)			
Unhydrolyzed urine	0.131 ± 0.033	21.4 ± 1.6	5.15 ± 0.24
Hydrolyzed urine	0.217 ± 0.014	765 ± 87	606 ± 11
Ratio of BB-22 3-carboxyindole in hydrolyzed urine to that in unhydrolyzed urine	1.65	35.7	118

Each concentration value is the mean ± standard deviation (SD) obtained from triplicate determinations

^a^Sample was unavailable

The urine levels of BB-22 of abusers have not been reported before. As listed in Table 4, the urine levels of BB-22 were determined to be 5.64, 5.52 and 6.92 pg/mL in the present cases 1, 2 and 3, respectively. Other SCs in urine were also detected previously by us with pg levels of this order. That is, the levels of six SCs were 10–232 pg/mL in [6], those of 5F-PB-22 of four abusers were 5.1–470 pg/mL in [7] and those of 5F-NNEI of two abusers were 5.1 and 7.9 pg/mL in [8].

In the present cases 1 and 2, serum and urine specimens were collected at nearly the same time. The relative ratios of BB-22 levels in urine to those in serum were calculated to be 0.032 in case 1 and 0.00096 in case 2, respectively, as shown in Table 4, indicating that much more sensitive detection is required for the quantification of SCs in urine than in serum.

The levels of BB-22 3-carboxyindole as the metabolite of BB-22 in human specimens also have not been reported before. The relative ratios of BB-22 3-carboxyindole levels in serum to BB-22 levels in serum were calculated to be 3.5 in case 1 and 7.6 in case 2, respectively, as shown in Table 4, indicating that the metabolite is the main component even in serum in these cases.

### Characterization of metabolites of BB-22 other than BB-22 3-carboxyindole in authentic urine specimens in cases 2 and 3

**Fig. 4:**
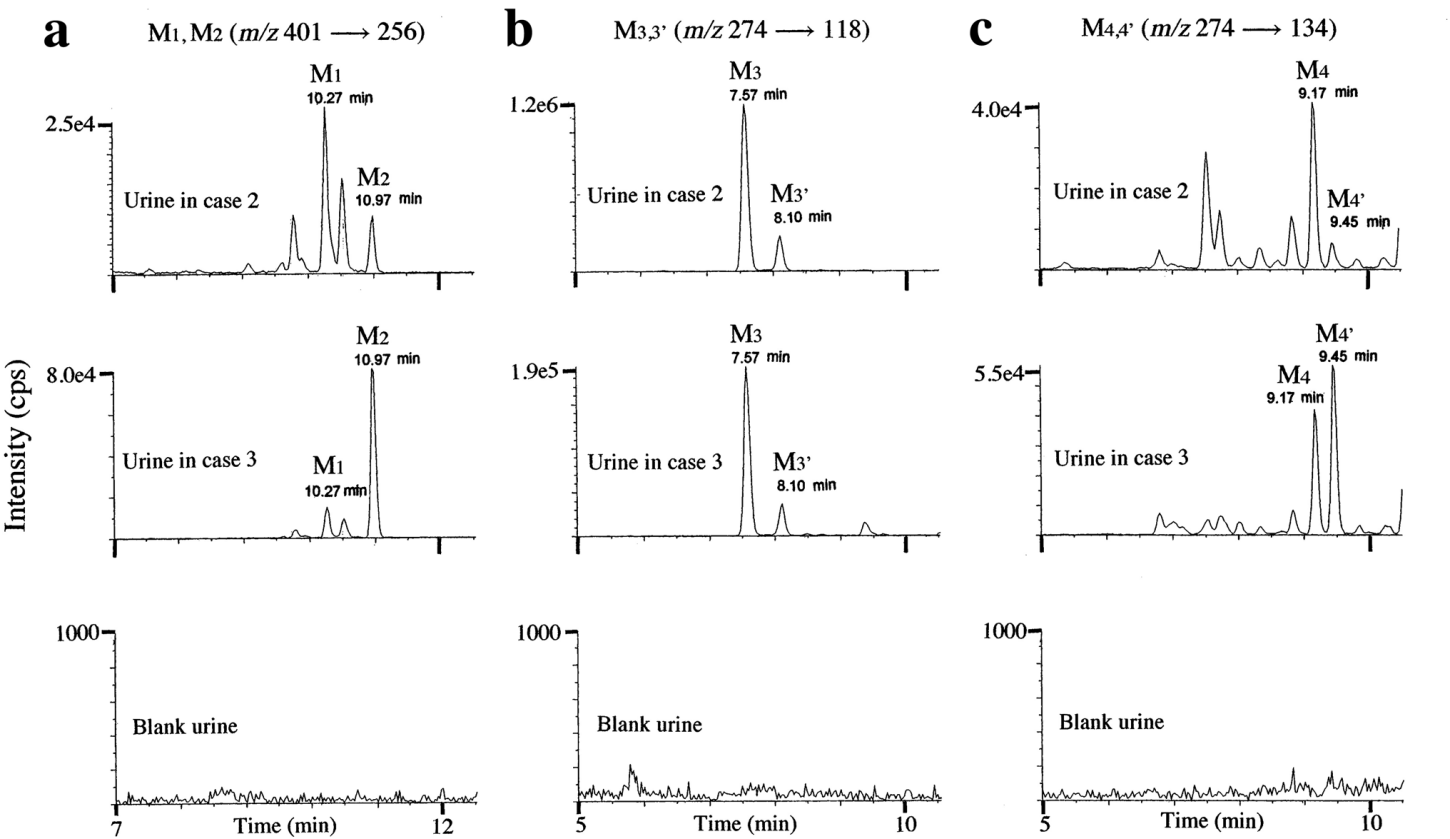
SRM chromatograms by LC–QTRAP-MS/MS for the detection of M1 and M2 (**a)**, where the extract from hydrolyzed urine in case 2, that in case 3 and that from blank urine are shown from the top to the bottom panel. The equivalent SRM chromatograms are also shown for the detection of M3,3′ (**b**) and M4,4′ (**c**). The collision energies for **a**, **b** and **c** were 21, 29 and 29 eV, respectively

Because the reference standards for metabolites of BB-22 other than BB-22 3-carboxyindole were not available, they could be identified only tentatively on the basis of high-resolution MS. As can be seen in Table 5, the positive protonated molecular metabolites could be measured down to the fourth decimal place with errors not greater than 2.0 ppm in many product ions using the high-resolution Orbitrap MS instrument. Both M1 and M2 metabolites were obtained by the ion transition of *m/z* 401 → 256, but could be differentiated by different retention times at 10.27 and 10.97 min, respectively (Fig. 4a). They were monohydroxyl metabolites of BB-22 at the cyclohexylmethyl and indole core moieties, respectively (Fig. 1).

**Table 5:** Accurate mass data, elemental composition, precursor ion in *m/z* with its mass error in parenthesis (ppm), diagnostic product ions in *m/z* with their mass errors in parentheses (ppm) for metabolites M1–M4′ in two cases

Metabolites	Elemental composition	Precursor ion in *m/z* observed (mass error, ppm)	Diagnostic product ions in *m/z* observed (mass errors, ppm)
M1	C_25_H_25_N_2_O_3_	401.1866 (− 1.3)	256.1340 (+ 3.1), 158.0605 (+ 3.2), 144.0443 (0), 130.0657 (+ 4.6), 95.0863 (+ 8.4)
M2	C_25_H_25_N_2_O_3_	401.1867 (+ 2.0)	256.1337 (+ 2.0), 160.0399 (+ 3.7), 97.1019 (+ 8.2), 55.0552 (+ 9.1)
M3,3′	C_16_H_20_NO_3_	274.1442 (+ 1.8)	256.1328 (− 6.6), 212.1436 (+ 1.4), 174.0556 (+ 4.0), 130.0653 (+ 1.5), 118.0653 (+ 5.9), 95.0859 (+ 4.2)
M4,4′	C_16_H_20_NO_3_	274.1438 (+ 0.4)	256.1334 (+ 0.8), 230.1535 (− 1.7), 192.0659 (+ 2.1), 148.0760 (+ 2.0), 134.0603 (+ 2.2), 97.1018 (+ 7.2)

Both M3 and M3′ were monohydroxyl metabolites formed from BB-22 3-carboxyindole at the cyclohexylmethyl moiety, with different retention times at 7.57 and 8.10 min, respectively (Fig. 4b). The exact locations of hydroxylation in the cyclohexylmethyl moiety were not known (Fig. 1). Both M4 and M4′ were also monohydroxyl metabolites formed from BB-22 3-carboxyindole at the indole core moiety with different retention times at 9.17 and 9.45 min, respectively (Fig. 4c). The exact locations of hydroxylation in the indole core moiety also could not be identified (Fig. 1).

**Fig. 5:**
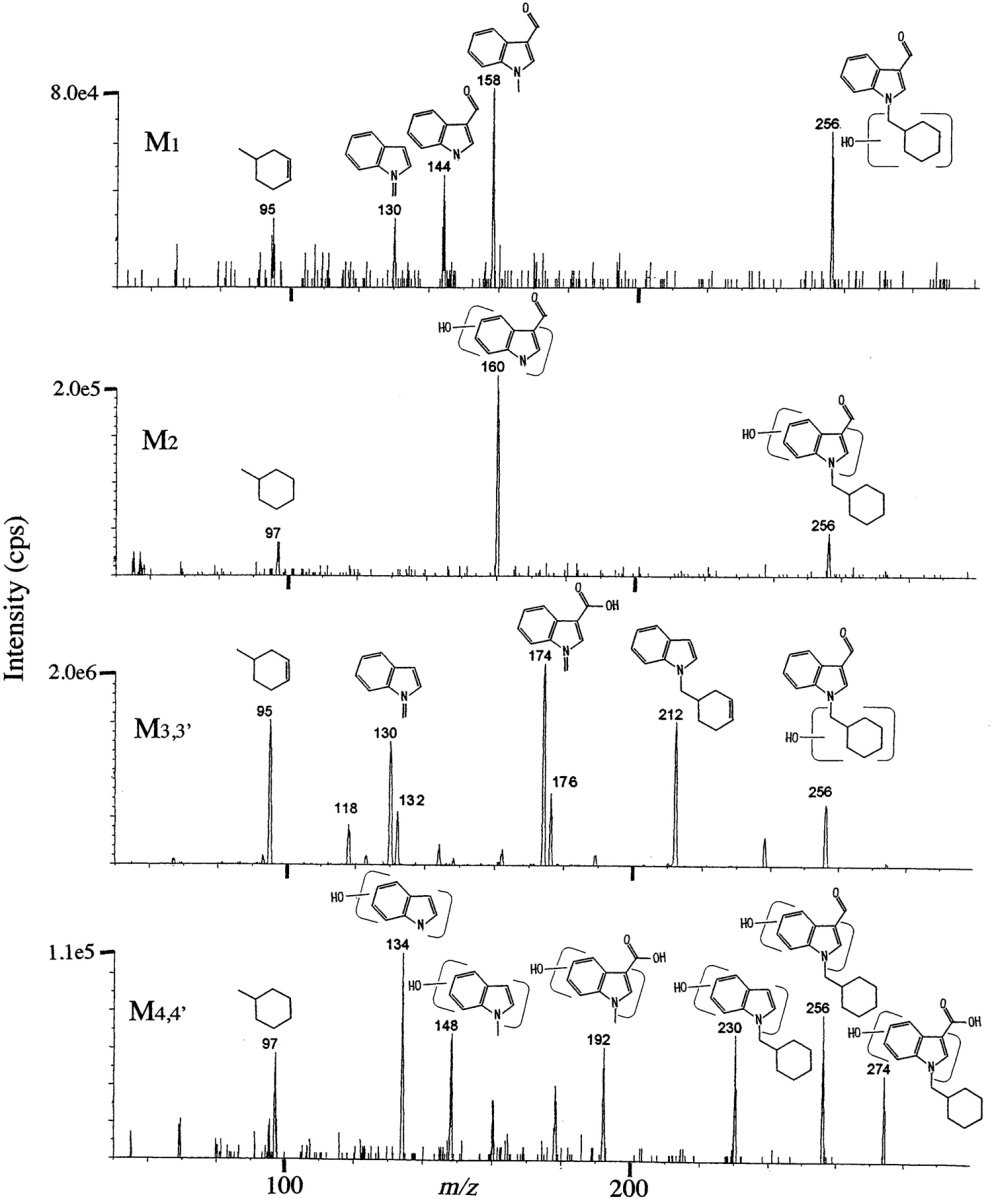
Product ion spectra by LC– MS/MS for the characterization of M1–M4,4′, where the extract from hydrolyzed urine in case 3 was used for M1 and M2 with collision energy at 51 eV and that in case 2 was used for M3,3′ and M4,4′ with collision energy at 29 eV

For confirmation of the structures of the above metabolites, their product ion spectra were measured by LC–MS/MS as shown in Fig. 5. The product ion spectrum of M3 and that of M3′ were almost the same; hence they are denoted as M3,3′, and for the same reason, the product ion spectrum of M4 and that of M4′ are denoted as M4,4′. Based on the elemental compositions of the product ions described in Table 5, the structures of major product ions were estimated as shown in Fig. 5, where only one structure was written for each product ion, although several regioisomers were possible for each product ion. The observed major product ions (Fig. 5) agreed quite well with the theoretical values measured by high-resolution MS as listed in Table 5.

In Fig. 5, all the metabolites M1–M4,4′ showed the diagnostic product ion at *m/z* 256 indicating that the monohydroxylation (i.e., + 16) occurred at BB-22 3-carboxylindole moiety showing its product ion at *m/z* 240 in Fig. 3a, b.

The product ion of M2 at *m/z* 160 indicated that the monohydroxylation occurred at indole core moiety of BB-22, whereas the product ion at *m/z* 97 indicated that the cyclohexylmethyl moiety of BB-22 showing its product ion at *m/z* 97 was intact. Therefore, monohydroxylation of M2 occurred at indole moiety.

The product ions of M4,4′ at *m/z* 230, 192, 148 and 134 were monohydroxylated ones of the ions at *m/z* 214, 176, 132 and 118, respectively, which were observed as the product ions of BB-22 3-carboxyindole in Fig. 3b, whereas the product ion of M4,4′ at *m/z* 97 indicated that its cyclohexylmethyl moiety was intact. Therefore, the monohydroxylation of M4,4′ occurred at indole moiety.

On the other hand, the product ion of M1 at *m/z* 95 suggested that the monohydroxylation occurred at cyclohexylmethyl moiety and then dehydration occurred there. The product ions of M1 at *m/z* 158 and 144 were the same product ions of BB-22 as observed in Fig. 3a, indicating that indole core moiety was not hydroxylated. The product ion of M1 at *m/z* 130 is considered to be the dehydrogenated ion of BB-22 3-carboxyindole at *m/z* 132, which is observable when hydroxylation-dehydration has occurred. Therefore, the monohydroxylation of M1 occurred at the cycohexylmethyl moiety.

The product ion of M3,3′ at *m/z* 95 indicated that the hydroxylation occurred at cyclohexylmethyl moiety and then dehydration occurred. The product ions of M3,3′ at *m/z* 176, 132 and 118 were the same product ions of BB-22 3-carboxyindole as observed in Fig. 3b, indicating that indole moiety was not hydroxylated. The product ions of M3,3′ at *m/z* 212, 174 and 130 were the dehydrogenated ions of BB-22 3-carboxyindole at *m/z* 214, 176 and 132, respectively, which is observable when hydroxylation-dehydration occurred. Therefore, the monohydroxylation of M3,3′ occurred at cyclohexylmethyl moiety.

In this way, the monohydroxylation at indole moiety and that at cyclohexylmethyl moiety of BB-22 as well as BB-22 3-carboxylindole have been clearly distinguished, although other regioisomers seem still to be included in each of the metabolites M1–M4,4′.

Because the quantifications of the metabolites M1–M4′ could not be realized, the relative peak height intensity ratios of each of them to the peak height of the IS at 1 ng/mL are presented in Table 6. The relative ratios of all the metabolites in the hydrolyzed urine were higher than those in the unhydrolized urine, indicating that they were in their conjugated forms, most probably glucuronidated forms.

**Table 6:** Relative intensities of metabolites M1–M4′ calculated as the ratio of its peak height to the peak height of the internal standard at 1 ng/mL in unhydrolyzed and hydrolyzed urine in two cases

Metabolite	Case	2	3
M1	Unhydrolyzed urine	0.00171 ± 0.00027	0.00073 ± 0.00012
	Hydrolyzed urine	0.242 ± 0.015	0.203 ± 0.022
M2	Unhydrolyzed urine	0.00099 ± 0.00016	0.00454 ± 0.00080
	Hydrolyzed urine	0.0787 ± 0.0042	1.20 ± 0.11
M3	Unhydrolyzed urine	1.050 ± 0.050	0.00727 ± 0.00074
	Hydrolyzed urine	12.4 ± 1.1	2.31 ± 0.16
M3′	Unhydrolyzed urine	0.209 ± 0.022	0.00089 ± 0.00024
	Hydrolyzed urine	2.16 ± 0.080	0.43 ± 0.065
M4	Unhydrolyzed urine	0.0204 ± 0.0020	0.0013 ± 0.00045
	Hydrolyzed urine	0.0320 ± 0.076	0.525 ± 0.019
M4′	Unhydrolyzed urine	0.00207 ± 0.00025	0.00243 ± 0.00053
	Hydrolyzed urine	0.0520 ± 0.0069	0.718 ± 0.022

Each value is the mean ± SD obtained from triplicate determinations

Although BB-22 3-carboxyindole, M3,3′ and M4,4′ were also produced from MDMB-CHMICA and ADB-CHMICA [5], M1 and M2 after hydrolysis are specific to BB-22. Therefore, the urinary concentrations of metabolites M1 and M2 after hydrolysis listed in Table 6 should be roughly compared with the urinary concentrations of BB-22 listed in Table 4. The peak height intensity ratio of 1 ng/mL of M1 or M2 to 1 ng/mL of IS can be roughly estimated to be 1 considering the structures of M1, M2 and IS. In that assumption, the concentrations of M1 and M2 will be 240 and 79 pg/mL in case 2, and those will be 200 and 1200 pg/mL in case 3, respectively. Therefore, the concentrations of M1 and M2 are calculated to be 41 and 13-fold higher than that of BB-22 in case 2, and 28 and 170-fold higher than that of BB-22 in case 3, respectively. This means that the detection of M1 and M2 in urine is easier than the detection of BB-22.

Very recently, Carlier et al. [5] have reported the in vitro metabolism of BB-22 by human hepatocytes. Although they have tentatively identified 10 metabolites, they did not include the M1 and M2 metabolites found in this in vivo study. The in vitro metabolites from the BB-22 3 carboxyindole were in common with those found in this study. It should be kept in mind that the metabolic pathways found in the in vitro experiments are not exactly the same as those found in urine specimens of drug users, despite that the in vitro hepatocytes are of human origin.

## Conclusions

To our knowledge, this is the first report to quantify BB-22 and BB-22 3-carboxyindole in authentic human urine specimens from three individuals. Furthermore, metabolites of BB-22 after hydroxylation (M1 and M2) and ester hydrolysis with hydroxylation (M3,3′ and M4,4′) have been characterized in urine specimens of two individuals for the first time by high-resolution-MS/MS and QTRAP-MS/MS.
